# *Nienna chukotka* sp. nov. (Protura, Acerentomidae, Nipponentominae) from the Arctic region, with a key to species of the genus

**DOI:** 10.3897/zookeys.899.47030

**Published:** 2019-12-12

**Authors:** Julia Shrubovych

**Affiliations:** 1 Institute of Soil Biology, Biology Centre, Czech Academy of Sciences, Na Sádkách 7, 370 05 České Budějovice, Czech Republic Institute of Soil Biology, Biology Centre, Czech Academy of Sciences České Budějovice Czech Republic; 2 Institute of Systematics and Evolution of Animals, Polish Academy of Science, Sławkowska 17, Pl 31-016 Krakow, Poland Institute of Systematics and Evolution of Animals, Polish Academy of Science Krakow Poland; 3 State Museum of Natural History, Ukrainian Academy of Sciences, Teatral’na St. 18, UA 79008 Lviv, Ukraine State Museum of Natural History, Ukrainian Academy of Sciences Lviv Ukraine

**Keywords:** Chaetotaxy, Chukotka, identification key, northern Palearctic, porotaxy

## Abstract

A new species of *Nienna* was collected in the most northern part of the Palearctic, inside the Arctic Circle. In possessing seta *Pc* on tergite VII and sternites VI–VII and a very long foretarsal sensillum *a*, *Nienna chukotka***sp. nov.** is more similar to *Alaskaentomon* species than to the other *Nienna* species distributed in southern Siberia and northern China. The new species differs from nearly all other members of Nipponentominae in possessing five anterior setae on tergite VII and in the presence of posterolateral pores on tergite I, as in members of *Hesperentomon* (Hesperentomidae). An identification key to *Nienna* species is provided.

## Introduction

The proturan genus *Nienna* Szeptycki, 1988 was created for *Nienna parvula* Szeptycki, 1988, described from the Altai mountains in southern Siberia (Szeptycki 1988). The genus differs from the 12 other genera of Nipponentominae Yin, 1983 in possessing a small, indistinctly granulated calyx and a short posterior filament on the maxillary gland, and in the small, nearly globular foretarsal sensillum *t3*. A second species, *Nienna quinghaiensis* Bu & Yin, 2008, was described from northern China. The diagnosis of the genus was recently updated (Galli et al. 2018). In the current paper, the description of a third species of *Nienna* is given. The type specimens, collected from the Arctic region, are the northernmost records for any Protura. A key to the species of *Nienna* is given.

## Materials and methods

Protura specimens collected from western Chukotka in 2018 were extracted from soil samples with Berlese-Tullgren funnels into 95% ethanol. The specimens were mounted on glass slides in Faure’s medium (Dunger and Fiedler 1989).

The classification system of Protura follows Szeptycki (2007). Terminology for body chaetotaxy and porotaxy follows Szeptycki (1988) and Shrubovych (2014); head seta designations follows Rusek et al. (2012).

### Abbreviations

**Abd.** abdominal segments,

**Th.** thoracic segments,

***A*-setae** anterior setae,

***P*-setae** posterior setae,

***fp*** frontal,

***cp*** clypeal,

***al*** anterolateral,

***sl*** sublateral,

***sal*** sternal anterolateral,

***psm*** posterosubmedial,

***psl*** posterosublateral,

***pl*** posterolateral,

***spm*** sternal posteromedial,

***spsm*** sternal posterosubmedial cuticular pore.

## Results

The genus *Nienna* is characterized by three pairs of *A*-setae on the mesonotum and metanotum, small, indistinctly granulated appendices on the calyx and a short posterior filament on the maxillary gland. The foretarsal sensillum *t1* is filiform, sensillum *t3* is small and globular (lanceolate in *N. quinghaiensis* Bu &Yin, 2008), the position of sensillum *d* is close to the base of *e*, and seta *β1* is setiform. Sensillum *a*’ is distal to the base of *t2.* Sensillum *b*’ is missing. The genus is similar to twelve other genera from the subfamily Nipponentominae in having abdominal legs with 2 nearly equal setae, 5 pairs of *A*-setae on tergites II–VI (except for *Alaskaentomon* Nosek, 1977 and *Nanshanentulus* Bu & Yin, 2007) and by the posterior position of seta *P3* on abdominal tergites II–VI (except for *A. fjellbergi* Nosek, 1977) (Bu and Yin 2007; Bu et. al. 2013; Galli et al. 2018; Nosek 1977, 1981; Shrubovych 2009, 2011, 2014; Shrubovych and Smykla 2012; Shrubovych et al. 2012; Shrubovych et al. 2014a, b, c).

### Nienna chukotka sp. nov.

Taxon classificationAnimaliaProturaAcerentomidae

41411DC6-9440-55FA-B1C5-3D0FA594C3F5

http://zoobank.org/2FEE913F-CC4C-445A-869E-F25B7267F644

**Figure 1.**
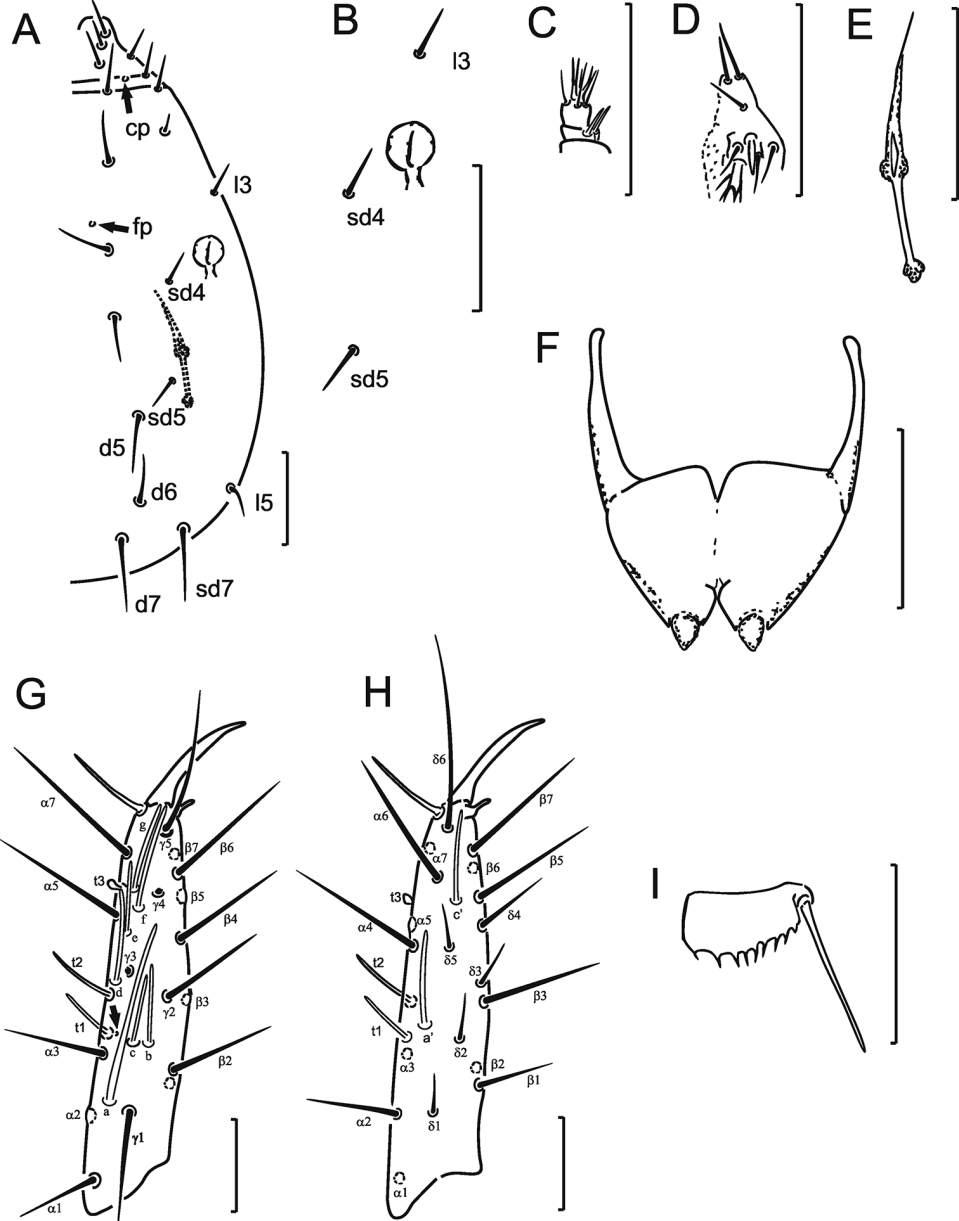
*Nienna chukotka* sp. nov. holotype. **A** Part of head **B** pseudoculus with setae *sd4*, *sd5* and *l3***C** maxillary palpus **D** labial palpus **E** maxillary gland **F** female squama genitalis **G** exterior view of foretarsus **H** interior view of foretarsus **I** comb. Arrows show pores. Scale bars: 20 µm.

**Figure 2.**
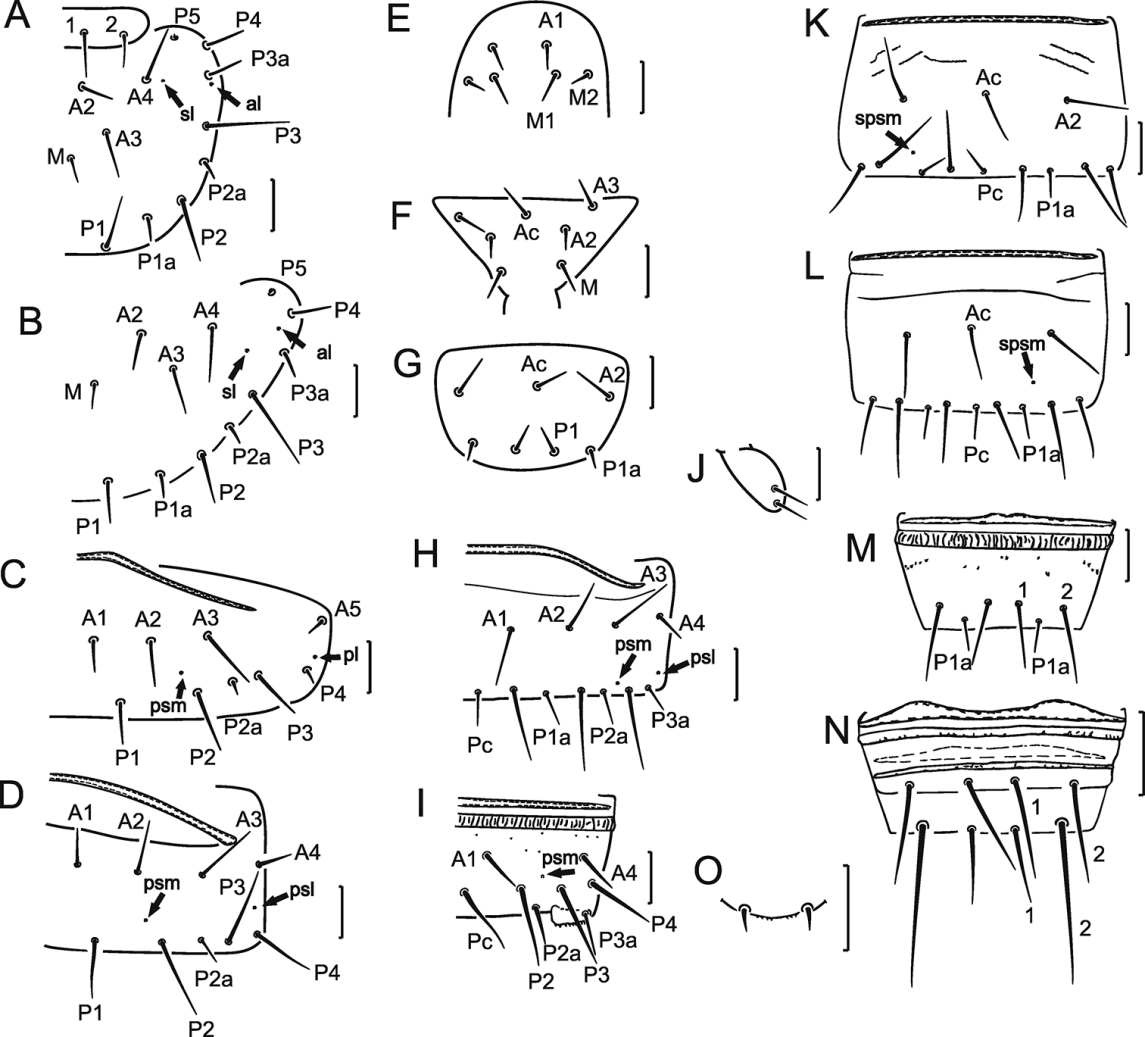
*Nienna chukotka* sp. nov. holotype. **A** Part of pro- and mesonotum **B** part of metanotum **C** part of tergite I **D** part of tergite VI **E** anterior part of prosternum **F** anterior part of mesosternum **G** sternite I **H** part of tergite VII **I** part of tergite VIII **J** abdominal leg of segment II **K** sternite VI **L** sternite VII **M** sternite VIII **N** sternites IX–X **O** hind margin of sternite XII. Arrows show pores. Scale bars: 20 µm.

Figs 1 , 2 , Table 1

#### Material examined.

Holotype (ISEA 6650): female, Russia, Chukotka Autonomous Okrug, Chaunskiy district, 2 kilometers from Apapelgino village, hill Akanotenmeem, in dry locality with *Dryas* sp., elev. 20 m, 69°48'40"N, 170°35'51"E, 24-VII-2018, coll. Makarov K. and Makarova O. Paratype (ISEA 6651): female, same data as holotype. The holotype and paratype are deposited in the collection of the Institute of Systematics and Evolution of Animals, Krakow, Poland (ISEA).

#### Diagnosis.

*Nienna chukotka* is characterized by 3 pairs of *A*-setae on the mesonotum, metanotum and tergite VIII, 3 *A*-setae on sternites I–VII, absence of *P1a* setae on tergites I–VI, 5 pairs of *A*-setae on tergites II–VII, absence of *A2* on prosternum, presence of seta *Pc* on tergite VII and sternites VI–VII, and presence of additional *d6* setae on head. Foretarsal sensillum *a* is broadened, very long, surpassing the base of sensillum *e.* Posterolateral pores (*pl*) present on tergite I, *psl* pores present on tergites VI and VII, asymmetrical *spsm* pores present on sternites IV–VII.

#### Description.

Head setae *l3*, *sd4* and *sd5* long, setiform, additional seta *d6* present, length ratio of posterior setae *d7*:*sd7:l5* as 2.4:2.5:1.0; frontal pore (*fp*) and a pair of clypeal (*cp*) pores present (Fig. 1A). Pseudoculus circular, with short posterior extension, PR = 12 (Fig. 1B). Sensilla of maxillary palps slender, pointed apically, equal in length (Fig. 1C). Labial palps with four-branched tuft of apical setae and broadened sensillum (Fig. 1D). Maxillary gland with small, indistinctly granulated calyx, short posterior filament and trilobed posterior dilation (Fig. 1E), CF = 6.0.

Foretarsus (Fig. 1J, H) without sensillum *b*’; *t1* filiform, *t3* small and globular; *a* broad, very long, evidently surpassing base of seta *γ3*, nearly reaching base of sensillum *f*; other sensilla parallel-sided. Sensillum *b* slightly longer than *c.* Sensillum *d* situated nearer to *e* than to *c*; *a*’ distal to level of *t2* insertion. Length formula of sensilla: *t3* < *t1* < *t2* < (*c* = *e*) < *b* < (*g* = *a’ = c*’) < (*d* = *f)* < *a.* Setae *β1* and *δ4* long and setiform, about twice as long as other *δ*-setae (Fig. 1H). Single pores situated near bases of sensilla *t1* (Fig. 1J) and *t3* (pore not visible on Fig. 1J because closed by sensillum *e*). Claw short, without inner tooth, empodial appendage short. BS = 0.4, TR = 2.7, EU = 0.3.

Formula of chaetotaxy given in Table 1. Setae on nota differing in length (Fig. 2A, B). Pronotal seta *1* 1.6 times longer than seta *2* (Fig. 1A). Meso- and metanota with setae *P1a* and *P2a* setiform, lengths 7 and 5 μm, respectively; *P2a* situated nearly midway between *P2* and *P3* (Fig. 2A, B). Length ratio of mesonotal setae *P1*: *P1a*: *P2* as 2.7: 1: 3.6. Meso- and metanota with *sl* and *al* pores (Fig. 2B). Pro-, meso- and metasterna without pores (Fig. 2E, F).

**Table 1.** Body chaetotaxy of *Nienna chukotka* sp. nov.

	**Dorsal**		**Ventral**	
**Segment**	**Formula**	**Setal composition**	**Formula**	**Setal composition**
Th. I	4	1, 2	(2+4)/6	A1, M1, 2,
				P1, 2, 3
Th. II	8/16	A2, 3, 4, M	(5+2)/4	Ac, 2, 3, M
		P1, 1a, 2, 2a, 3, 3a, 4, 5		P1, 3
Th. III	8/16	A2, 3, 4, M	(7+2)/4	Ac, 2, 3, 4, M
		P1, 1a, 2, 2a, 3, 3a, 4, 5		P1, 3
Abd. I	8(6)/10	A1, 2, (3), 5	3/4	Ac, 2
		P1, 2, 2a, 3, 4		P1, 1a
Abd. II-III	10/14	A1, 2, 3, 4, 5	3/5	Ac, 2
		P1, 2, 2a, 3, 4, 4a, 5		Pc, 1a, 2
Abd. IV-V	10/14	A1, 2, 3, 4, 5	3/8	Ac, 2
		P1, 2, 2a, 3, 4, 4a, 5		P1, 1a, 2, 3
Abd. VI	10/14	A1, 2, 3, 4, 5	3/9	Ac, 2
		P1, 2, 2a, 3, 4, 4a, 5		Pc, 1, 1a, 2, 3
Abd. VII	10/19	A1, 2, 3, 4, 5	3/9	Ac, 2
		Pc, 1, 1a, 2, 2a, 3, 3a, 4, 4a, 5		Pc, 1, 1a, 2, 3
Abd. VIII	6/15	A1, 4, 5	4/2	1, 2
		Pc, 2, 2a, 3, 3a, 4, 4a, 5		P1a
Abd. IX	12	1, 1a, 2, 2a, 3, 4	4	1, 2
Abd. X	10	1, 2, 2a, 3, 4	4	1, 2
Abd. XI	6	1, 3, 4	6	
Abd. XII	9		6	

(3) – setae *A3* absent in paratype. Tergite I with 6 *A*-setae.

Accessory setae on tergites and sternites I–VII setiform, those of tergite VII significantly longer than those on I–VI. (Fig. 2C, D, G, H, K, L). Pores *pl* present on tergite I, *psm* on tergites I–VII, *psl* on tergites VI–VII, *al* on tergites II–VII (Fig. 2C, D, H).

Abdominal legs with 4, 2, 2 setae. Subapical and lateral apical setae on second and third pairs of abdominal legs nearly equal in length, 15 and 14 μm, respectively (Fig. 2J). Sternites I–III without pores (Fig. 2G). Sternites IV–VII with asymmetrical *spsm* pore, with short anterolateral lines and sternite VII with a connecting line on anterior part (Figs 2K, L).

Abdominal segment VIII with distinct striate band; tergite and sternite anteriorly with irregular small teeth (Figs 2 I, M). Pore *psm* without accompanying teeth. Posterior margin of sternite VIII and laterotergites smooth. Comb VIII with 9–10 small teeth (Fig. 1I). Seta *1a* on tergite IX half the length of seta *1*. Seta *2a* on tergites IX and X shorter than other setae. Sternites IX–X with traces of striate band (Fig. 2N). Setae *1* and *2* on sternite IX of equal length, on sternite X seta *1* about half the length of seta *2* (Fig. 2N). Medial pore on dorsal lobe of segment XII and pair of *sal* pores on ventral lobe. Hind margin of dorsal lobe smooth, ventral lobe with fine serrations (Fig. 2O).

Female squama genitalis with short, pointed acrostyli (Fig. 1F).

Body measurements (2 females) (in μm): maximum body length 1004, head 115, pseudoculus 8, lever 3, posterior part of maxillary gland 12; pronotal setae *1* 18, *2* 11; mesonotal setae *P1* 19, *P1a* 7, *P2* 25, *M* 10, foretarsus 94–95, claw 30, empodial appendage 4.

#### Chaetal variability.

In the holotype, seta *P4* is doubled asymmetrically on the mesonotum; in the paratype, seta *A3* is absent symmetrically on tergite I and seta *P2a* is doubled on tergite VII.

#### Etymology.

The species name is taken from the general locality where the specimens were collected.

#### Remarks.

*Nienna chukotka* sp. nov. differs from *N. parvula* and *N. quinghaiensis* in the presence of seta *Pc* on tergite VII and sternites VI–VII (in *N. quinghaiensis* seta *Pc* is present on sternite VII only), the presence of 5 pairs of *A*-setae (4 pairs in the other two species) and *P3a* on tergite VII, the shape of the accessory setae on tergites and sternites I–VI (setiform in the new species, sensilliform in the other two species) and the shape of foretarsal sensilla *a*, *c* and *e* (in the other species sensillum *a* is shorter, reaching base of sensillum *t2*, sensilla *c* and *e* short and broad). The porotaxy of meso- and metanota and abdominal sternites also differs: *Nienna chukotka* has two pairs of *sl* and *al* pores on the meso- and metanota, and asymmetrical *spsm* pores on sternites IV–VII;], whereas *N. parvula* has a pair of *sl* pores on the meso- and metanota, and a simple *spm* pore on sternites VI–VII. *Nienna quinghaiensis* has *al* and *l* pores on the mesonotum, *l* pores on the metanotum, and an *spm* pore on sternite VII. The new species is more similar to *N. parvula* in possessing traces of a striate band on sternites IX–X and in the globular foretarsal sensillum *t3*. *Nienna chukotka* is characterized by the presence of *pl* on tergite I, which is the first report of posterolateral pores in Acerentomidae. Szeptycki (1988) previously described *pl* pores on *Hesperentomon martynovae* Szeptycki, 1988 (Hesperentomidae) collected in the Altai Mts. These *pl* pores have also been recorded in other *Hesperentomon* species: *H. fopingense* Bu, Shrubovych & Yin, 2011, *H. nanshanensis* Bu & Yin, 2007, *H. xiningense* Bu & Yin, 2007 distributed in China, and *H. tianshanicum* Martynova, 1970 (Shrubovych 2010).

## Discussion

The foretarsus of *N. chukotka* sp. nov. has a very long sensillum *a*, surpassing the base of sensillum *e*, and filiform foretarsal sensillum *t1*, characters shared with two species of *Alaskaentomon* (*A. fjellbergi*, *A. condei*). These two *Alaskaentomon* species possess seta *Pc* on tergite VII and sternites VI–VII. *Alaskaentomon* spp. differ from *N. chukotka* sp. nov. in having two pairs of *A*-setae on the meso- and metanota and large granulated appendices on the calyx of the maxillary gland (Shrubovych et al. 2014c). In notal chaetotaxy (three pairs of *A*-setae) and the filiform sensillum *t1*, the genus *Nienna* is similar to the genera *Callientomon* Yin, 1980, *Noldo* Szeptycki, 1988, *Paracerella* Imadaté, 1980 and *Verrucoentomon* Rusek, 1974. However, *Nienna* differs from all of them in possessing small, indistinctly granulated appendices on the calyx of the maxillary gland and in the small, nearly globular foretarsal sensillum *t3* (Shrubovych et al. 2014a). The new species differs from nearly all species of Nipponentominae in possessing a pair of *A1* setae on tergite VII (five pairs of *A*-setae), while nearly all other nipponentomines have four pairs of *A*-setae (except *Nipponentomon macleani* Nosek, 1977 from Alaska, which also has 5 pairs of *A*-setae). Therefore, *N. chukotka* sp. nov. from Chukotka is more similar in body chaetotaxy and in foretarsal sensilla pattern to members of other genera distributed in Alaska than to the other *Nienna* species distributed in more southern regions of the Palearctic. This peculiarity could be an effect of subsequent allopatric speciation resulting from successive closings of the Bering Strait and cooling of the Arctic Ocean during the Pliocene-Pleistocene. Another interesting fact is that species recorded on the northern edge of proturan distribution (only a few Protura species are known from the Arctic region) possess a larger number of setae on the body than species with a more southern distribution.

### Key to *Nienna* species

**Table:** 

1	Foretarsal sensillum *a* very long, surpassing base of sensillum *e*, tergite VII with 5 pairs of *A*-setae and with *Pc*, *P3a* present, sternites VI–VII with *Pc*	***Nienna chukotka* sp. nov.**
–	Foretarsal sensillum *a* short, nearly reaching base of sensillum *t2*, tergite VII with 4 pairs of *A*-setae and without *Pc*, *P3a* absent, sternite VI without *Pc*, sternite VII with or without *Pc*	**2**
2	Foretarsal sensilla *b* and *c* nearly equal in length, seta *δ4* setiform, sternite VII without *Pc*	***N. parvula* Szeptycki, 1988**
–	Foretarsal sensillum *c* half the length of *b*, seta *δ4* sensilliform, sternite VII with *Pc*	***N. quinghaiensis* Bu & Yin, 2008**

## Supplementary Material

XML Treatment for Nienna chukotka
